# Analytical Validation of a Highly Quantitative, Sensitive, Accurate, and Reproducible Assay (HERmark**^®^**) for the Measurement of HER2 Total Protein and HER2 Homodimers in FFPE Breast Cancer Tumor Specimens

**DOI:** 10.4061/2010/814176

**Published:** 2010-06-28

**Authors:** Jeffrey S. Larson, Laurie J. Goodman, Yuping Tan, Lisa Defazio-Eli, Agnes C. Paquet, Jennifer W. Cook, Amber Rivera, Kristi Frankson, Jolly Bose, Lili Chen, Judy Cheung, Yining Shi, Sarah Irwin, Linda D. B. Kiss, Weidong Huang, Shannon Utter, Thomas Sherwood, Michael Bates, Jodi Weidler, Gordon Parry, John Winslow, Christos J. Petropoulos, Jeannette M. Whitcomb

**Affiliations:** ^1^Department of Clinical Laboratory Operations, Monogram Biosciences, Inc., South San Francisco, CA 94080, USA; ^2^Department of Oncology, Research and Development, Monogram Biosciences, Inc., South San Francisco, CA 94080, USA; ^3^Department of Clinical Research, Monogram Biosciences, Inc., South San Francisco, CA 94080, USA

## Abstract

We report here the results of the analytical validation of assays that measure HER2 total protein (H2T) and HER2 homodimer (H2D) expression in Formalin Fixed Paraffin Embedded (FFPE) breast cancer tumors as well as cell line controls. The assays are based on the VeraTag technology platform and are commercially available through a central CAP-accredited clinical reference laboratory. The accuracy of H2T measurements spans a broad dynamic range (2-3 logs) as evaluated by comparison with cross-validating technologies. The measurement of H2T expression demonstrates a sensitivity that is approximately 7–10 times greater than conventional immunohistochemistry (IHC) (HercepTest). The HERmark assay is a quantitative assay that sensitively and reproducibly measures continuous H2T and H2D protein expression levels and therefore may have the potential to stratify patients more accurately with respect to response to HER2-targeted therapies than current methods which rely on semiquantitative protein measurements (IHC) or on indirect assessments of gene amplification (FISH).

## 1. Introduction

The human epidermal growth factor receptor 2 (HER2) is a transmembrane protein tyrosine kinase receptor that is important in initiating signal transduction pathways in normal and abnormal cells [1–5]. HER2 is overexpressed/amplified in approximately 15%–30% of human breast tumors and is a biomarker of poor prognosis in patients demonstrating either high protein levels and/or gene amplification on chromosome 17 [4, 6, 7]. For this reason, HER2 testing is recommended for all newly diagnosed breast cancer patients for the selection of individuals that may benefit from treatment with the humanized monoclonal antibody Trastuzumab [8–11]. Despite confirmed overexpression of HER2, the current response rates to Trastuzumab are less than 50% in the metastatic setting and many of the patients that respond initially will eventually develop resistance and subsequent recurrence of their disease [12–14]. Standardization of both IHC and ISH methodologies across laboratories remains a major problem [15]. because of this approximately 20% of HER2 testing performed in the field may be inaccurate [16]. The ability to accurately and reproducibly quantify the level of HER2 protein expression in tumors is critical to the appropriate selection of patients for Trastuzumab and other HER2 targeted therapies. 

Most laboratories in North America and Europe use IHC to determine HER2 protein status, with equivocal category results confirmed by indirectly measuring HER2 gene amplification by Fluorescence *In Situ* Hybridization (FISH) or more recently by the Chromogenic *In Situ* Hybridization (CISH) assay [15, 17]. The College of American Pathology Guidelines for HER2 testing algorithm includes testing all newly diagnosed breast cancer patients. Patients with tumors that are classified as >30% 3+ by IHC or FISH positive (>2.2 HER2/CEP17 or >6 for noncorrected) are considered HER2 positive and eligible for treatment with Trastuzumab, while those that are IHC 2+ should be further confirmed by FISH testing [15, 18]. If the tumors that were originally categorized as IHC 2+ are confirmed to be FISH positive these patients become eligible for Trastuzumab treatment [10, 18, 19]. These routine tests are subject to interlaboratory variation in both the accuracy and reproducibility between these two methods with a general concordance rate published for many studies of approximately ~80%–90% (excluding IHC 2+ cases) for IHC and FISH, well below the 95% threshold mandated by the new ASCO-CAP guidelines [20]. Furthermore, a recent study was undertaken to evaluate concordance between local and central laboratory testing for HER2 in breast cancer specimens from the North Central Cancer Treatment Group, N9831 Intergroup Adjuvant Trial. The results demonstrated that the concordance rate between local and central laboratory HER2 testing was between 75%–82% depending on whether which IHC technique was used [21]. It is therefore critical that new testing methods that are less subjective and more quantitative, accurate, and reproducible to improve our ability to appropriately select patients for expensive HER2 targeted therapies. There are two FDA approved tests for IHC, HercepTest (Dako, Carpentaria, CA), and Pathway (Ventana Medical Systems Inc, Tucson, AZ), both of which utilize a single antibody format on standard thin section FFPE tumors and the results are based on a subjective, semi-quantitative scale (0, 1+, 2+, 3+) requiring microscopic evaluation and scoring by a board certified pathologist [21]. There are four tests that are FDA approved for FISH/CISH testing, including PathVysion (Vysis Inc, Downers Grove, IL), INFORM (Ventana Medical Systems Inc.), HER2 FISH pharmDx (Dako), and the SPoT-Light CISH kit (Invitrogen, Carlsbad, CA) [21, 22]. FISH/CISH testing is also semiquantitative and subject to inter-laboratory differences accounting for the relatively low (88%) concordance rates demonstrated between local and central FISH testing [21]. Because of these statistics, there can be a number of false positive and negative results from the use of these methods resulting in the inappropriate and the cost-inefficient treatment of patients with Trastuzumab.

To address the need for better methodologies of HER2 testing, we have developed an assay that can quantitatively measure HER2 protein levels as well as the functional HER2:HER2 homodimers in FFPE breast tumor specimens. The assay has been validated according to the standards defined by CLIA and is routinely performed in a CAP-accredited clinical reference laboratory. The assays are based on the VeraTag technology platform [23] which utilizes a proximity-based release of a fluorescent tag bound to a specific antibody and the subsequent quantification of this tag by capillary electrophoresis. In the most common format of the platform, the dual-antibody approach provides significantly increased specificity and sensitivity compared to single antibody-based IHC methods. The method provides a continuum of highly quantitative results that span the entire dynamic range of HER2 protein expression from 0 to 3+ if compared with conventional IHC scoring methods, thereby providing a more accurate assessment of the HER2 status of a patient tumor. 

In addition to the quantitative advantage of the VeraTag assay, results obtained with HERmark assay from a clinical cohort of 237 metastatic breast cancer patients originally classified by a combination of IHC and FISH, demonstrated 98% concordance for IHC positive and negative assay values (excluding equivocals) [24] This is well within the ASCO/CAP guidelines requiring laboratories to show at least 95% concordance with another validated test for negative and positive assay values [15]. The overall concordance of the recent HER2 mRNA testing included as part of the Oncotype Dx report is 70%–78% when compared with central IHC [25]. The concordance of HER2 mRNA measurements with central FISH is higher (97%) but this makes sense when considering that both are indirect measurements of drug response. Taken together, this supports the need for quantitative and reproducible measurements the actual drug target rather than relying on indirect measurements such as gene amplification or mRNA levels or with subjective quantitation by IHC. An additional advantage of the VeraTag platform is the ability to quantitatively measure protein-protein interactions and specifically one of the primary activated or functional forms of the HER2 protein (homodimer/proximer) [23] and potentially the true drug target. The HERmark assay is a quantitative assay that accurately and reproducibly measures HER2 total and homodimer protein expression on a continuum as opposed to the subjective classification criteria of the current FDA approved HER2 IHC protein assays and therefore may have the potential to stratify patients quantitatively for response to Trastuzumab as well as other targeted therapies that are making their way into the clinic.

## 2. Material and Methods

### 2.1. Antibodies, Isotype Controls, Photosensitizer Molecule, and Illumination Buffer

Monoclonal antibodies Ab8 and Ab15 directed against the intracellular domain of HER2 were purchased from Lab Vision (Fremont, CA). Mouse monoclonal antihuman IgG_1_ isotype control antibodies, biotinylated and unconjugated, were purchased from BD Biosciences (Franklin Lakes, NJ). The fluorescent reporter Pro11 was synthesized and purified according to protocol described earlier (US Patent 7,105,308). Antibody-fluorescent tag and antibody-biotin conjugates Ab8-Pro11, Ab8-biotin, Ab15-biotin, and IgG_1_-Pro11 were conjugated and purified as described previously [23]. Ab8-Pro11 and IgG_1_-Pro11 were matched to have similar hapten ratios. After purification, all antibodies were stored in 1X PBS with 1 mg/mL BSA and 0.001% sodium azide (Sigma-Aldrich, St. Louis, MO). Streptavidin-conjugated methylene blue (SA-MB: “photoactivator molecule”) was synthesized and purified according to the protocol described earlier (US Patent 7,105,308). Illumination Buffer (IB) contained 3 pM fluorescein and 2 additional capillary electrophoresis (CE) mobility markers in 0.01X PBS. The fluorescein was used as a quantitation standard.

### 2.2. Illuminator and Chiller Blocks, CE Instruments, and Slide Scanner

Three LED array illuminators customized with chiller blocks (Torrey Pine Scientific, San Diego, CA) were used to release reporter tags. Three ABI 3130 genetic analyzer CE instruments equipped with 22 cm arrays (Applied Biosystems, Foster City, CA) were used to detect VeraTags. An HP ScanJet 4890 flatbed scanner was used to create a digital image of hematoxylin and eosin (H & E) stained slides. Section area (mm^2^) was measured using ImageJ software as described previously [23].

### 2.3. Cell Lines, Tissues, Cell Culture, Fixation, Processing and Paraffin Embedding

The following breast cancer cell lines were obtained from the American Type Culture Collection (ATCC, Manassas, VA): SkBR3, BT-20, MDA-MB-453, MCF-7, MDA-MB-468, BT-474, MDA-MB-361, T47D, MDA-MB-231, Zr75-1, and A431. One melanoma cell line was utilized MDA-MB-435S [26]. A431, MDA-MB-231, MDA-MB-361, MDA-MB-453, and MDA-MB-468 were maintained in Dulbecco's Modified Eagle Medium (DMEM). BT-474 and MDA-MB-435S were maintained in Dulbecco's Modified Eagle Medium: Nutrient Mixture F12 (DMEM/F12). T47D and Zr75-1 were maintained in RPMI Media 1640. MCF-7 cells were maintained in Minimum Essential Medium Alpha (*α*MEM). BT-20 cells were maintained in Eagle's Minimum Essential Medium with Earle's Balanced Salt Solution (EMEM + EBSS). SkBR-3 was maintained in McCoy's 5 A media. All of the media were supplemented with 10% fetal bovine serum (FBS), 10 mM penicillin-streptomycin, and 10 mM Glutamax. All media and supplements were purchased from Invitrogen (Carlsbad, CA). All cell lines were cultured in a 37°C humidified atmosphere containing 95% air and 5% CO_2_ and were split and media replenished according to ATCC recommendations. Cells were screened for Mycoplasma contamination using standard PCR methods. Forty-six patient derived tumors were purchased as frozen tumor sample or tumor blocks from Asterand (Detroit, MI), William Bainbridge (Seattle, WA), or Proteogenix (Costa Mesa, CA). Frozen breast tissues were made into FFPE blocks as previously described [23]. Two tumor tissues were used to test the effect of interfering substances by being embedded with pathologically verified normal breast stroma and fat samples.

After the cell cultures reached appropriate confluence, the cells were harvested 10–18 hours after being refed with the appropriate media. Media was removed from the 500 cm^2^ plates and the cells were washed with 1X PBS. The 1X PBS was aspirated and approximately 25 mL of Richard-Allan Pen-Fix Formalin Fixative (Thermo Fisher Scientific, Waltham, MA) was added to the cells. Cells were scraped and fixed overnight at 4°C. Cells were then processed into FFPE blocks as previously described [23]. Cell lines and tumor tissue FFPE blocks were cut at 7 *μ*m and 5 *μ*m thickness, respectively, using a Leica RM 2145 and 2155 Rotary Microtome (Leica Microsystems, Bannockburn, IL). Sections were placed on positively charged glass slides (VWR, West Chester, PA), air dried for 30 minutes during the time of cutting all the sections, and then baked at 60°C for 1.5 hours. All slides were stored at 4°C until assayed and generally used within 3-4 weeks.

## 3. ELISA, Flow Cytometry, and IHC

Lysates were prepared from 500 cm^2^ plates at 80% confluency. Cells were collected by scraping and washed three times with cold PBS. The resulting pellets were lysed in buffer containing 1% Triton X-100, 50 mM Tris, pH 7.5, 50 mM NaF, 50 mM *β*-glycerophophate, 100 mM NaCl, 1 mM Na3VO4, 100 *μ*g Pepstatin A, 5 *μ*m EDTA (Sigma-Aldrich, St. Louis, MO), and 1 complete mini protease inhibitor tablet (Roche, Basel, Switzerland). After lysis the samples were centrifuged for 20 minutes at 4°C and the supernatants were stored at −80°C. Protein content for each lysate was determined against a standardized control using the Pierce Protein Assay Kit (Thermo Fisher Scientific, Waltham, MA). Determination of HER2 protein content was performed with a commercially available quantitative enzyme-linked immunosorbent assay (ELISA) kit (EMD Biosciences, San Diego, CA) according to the manufacturer's protocol. Cell lysates were serially diluted and measured in triplicate. Each lysate was tested on three different days. HER-2 protein levels were determined in nanogram per milligram of total protein (Table 1). For flow cytometry analysis, cells were harvested by trypsinization and counted. 5 × 10^5^ cells were placed in a 96 well plate and labeled with biotinylated primary monoclonal mouse antihuman ErbB2 Ab4 (Lab Vision, Fremont, CA) at a concentration of 4 *μ*g/ml in 100 *μ*l total volume. Isotype controls were run using mouse IgG_1_ (BD Biosciences, Franklin Lakes, NJ). The cells were incubated with antibody on ice for 45 minutes. Postincubation, the cells were washed twice with 1X PBS, followed by labeling with R-Phycoerythrin (PE)-Avidin (Invitrogen, Carlsbad, CA) at 2 *μ*g/ml for 30 minutes on ice. The labeled cells were washed with 1X PBS twice and fixed with 1% paraformaldehyde in 1X PBS. FACS analysis was performed on a FACSCalibur cytometer (BD Biosciences, San Jose, CA). PE fluorescence intensity of labeled cells was determined on a FL2 (585/42 nm band pass filter) detector. The direct quantitation of the fluorescence intensity of samples in terms of number of molecules of ErbB2 receptors was generated based on a calibrated standard curve using Quantum PE MESF Kit (Bangs Laboratories, Inc, Fishers, IN). IHC was performed on Discovery XT automated staining system (Ventana Medical Systems, Tucson, AZ) using Ventana reagents according to the manufacturers suggested protocol with slight modifications. Briefly, slides were labeled, deparaffinized and the Discovery XT was programmed to perform cell conditioning using CC1 buffer. After blocking, sections were incubated for 1 hour with CB11 (Ventana cat # 760-2694), rinsed, then incubated with secondary antibody for 32 minutes. After DAB detection, the sections were counterstained with hematoxylin followed by bluing reagent. The slides were removed, rinsed with PBST and dehydrated using ethanol/xylene and coverslips applied for microscopic evaluation.

## 4. HERmark Assay

A batch of samples consists of 3 normalization controls, spanning the dynamic range of the assay, 1 negative control, 1 accuracy control, and 15 patient tumor samples. FFPE samples were loaded into a slide rack and placed in a container filled with xylenes where they soaked for five minutes, being agitated slightly. The samples were moved to a fresh container of xylenes for an additional five minutes. These steps were repeated with 100% reagent alcohol, 70% ethanol, and finally deionized water. Immediately after deparaffinization, the samples were placed in Diva Decloaker (BioCare Medical, Concord, CA), a citrate-based buffer. Heat-induced epitope retrieval was performed in a Decloaking Chamber (BioCare Medical, Concord), a pressure cooker, set at 95°C for 40 minutes. After cooling for 1 hour and rinsing with water, a hydrophobic circle was drawn on the slide to retain reagents in a defined area. Sections were blocked as previously described [23]. After blocking, samples were incubated with 4 *μ*g/mL of Ab8-Pro11 and 4 *μ*g/mL Ab15-biotin for the H2T assay and 2 *μ*g/mL of Ab8-Pro11 and 2 *μ*g/mL Ab8-Biotin for the H2D assay overnight in a humidified chamber at 4°C. Post incubation, samples were rinsed and incubated with Strepavidin-methylene blue (SA-MB) as previously described [23]. Illumination buffer (IB) was added to samples and slides were transferred to the illuminator/chiller block. VeraTag Reporters were released at approximately 4°C as previously described [23]. Post illumination, slides were removed from the chiller-block and incubated in a humidified chamber at RT for 1 hour. Samples were collected from the slides and reduced in a final concentration of 5 ng/mL of sodium borohydride. Subsequently, samples were diluted 1 : 5 and both neat and diluted samples were separated and detected on an ABI 3130 capillary electrophoresis (CE) instrument. The injection parameters were 6 kV, 100 s at 30°C and 6 kV, 50 s at 30°C, for the H2D and H2T assays, respectively. Upon completion of the HERmark assay, samples were immediately H & E stained using standard protocols and reagents. A board certified pathologist using standard criteria for invasive carcinoma determined and circled the tumor area. Circled slides were scanned and tumor area was calculated using the digital image of the sample. The program used to quantify the image was Image-Pro Plus version 6.0.0.260 (Media Cybernetics, Inc.; Bethesda, MD).

## 5. Data Analysis

Data analysis was performed as previously described [23]. In brief, signals from two concomitantly run CE markers are used to demarcate the relevant region of the electropherogram and to locate the assay-specific peaks from the VeraTag reporters. Once identified, the signal intensity is calculated for each VeraTag reporter as the peak height integrated over the peak elution time. The VeraTag peak area is then normalized to the peak area of the internal standard fluorescein, resulting in the relative peak area (RPA), which is proportional to the concentration of the HER2 analyte being measured. The RPA is then normalized to volume of illumination buffer (IB) and tumor area (mm^2^) (TA) using this calculation: RPA*IB/TA.

Finally, reported values were adjusted to reflect batch-to-batch trending in assay performance. Controls and samples were normalized by multiplying their adjusted RPA with the calculated Batch Normalization Factor (BNF). Adjusted RPAs of the controls within a batch were compared to reference values. A nonlinear regression analysis on the paired data was performed and a BNF determined for each batch. Separate BNFs were determined for the released peak and the converted peak. Each adjusted RPA value was multiplied by the respective BNF to obtain a normalized RPA value. Because this normalized RPA value was unit-less by definition, its value was referred to in *VeraTag* units. All reportable normalized values for both peaks for a given sample were averaged to determine a final value for that sample. 

An algorithm was generated so that individual data points within each validation parameter could be compared in a pairwise fashion. Because a single patient sample is run for each of the H2T and H2D assays, performing pairwise comparisons allows for a high degree of confidence in that single measurement.

## 6. Results

### 6.1. Configuration of the H2T and H2D Assays

**Figure 1:**
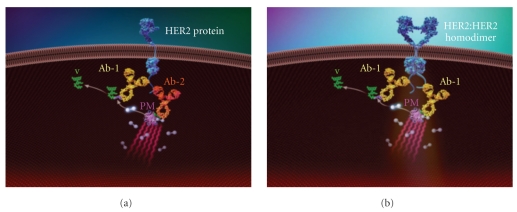
*Configuration of the H2T and H2D assay.* (a) Configuration of the HER2 total assay consists of two antibodies specific to unique epitopes on the c-terminus of the HER2 protein. One antibody (Ab-1) is conjugated to a VeraTag molecule (V) and the other is conjugated to biotin (Ab-2). The photosensitizer molecule (PM) brings the strepavidin-methylene blue in close proximity to the antibodies. (b) Configuration of the H2D assay consists of one antibody that is specific for a unique epitope on the HER2 protein. The same antibody is conjugated either to a VeraTag (V; Ab-1) or to biotin (Ab-1). The photosensitizer molecule (PM) brings the strepavidin-methylene blue in close proximity to the antibodies.

The principles of the VeraTag technology have been published elsewhere [23]. In brief, the HER2 total assay configuration (Figure 1(a)) consists of a VeraTag reporter conjugated to a specific antibody (Ab-1) that binds a distinct epitope of HER2, while biotin is conjugated to a second antibody (Ab-2) that recognizes a second unique HER2 epitope. In a subsequent reaction, a secondary streptavidin-methylene blue conjugate is bound to the biotin-antibody complex to form a photosensitizer molecule (PM). Upon photoactivation, the PM allows the generation of reactive oxygen that subsequently cleaves the most proximal VeraTag reporter, yielding a released fluorescent molecule with a distinct charge to mass ratio. The fluorescent molecule is accurately quantified using standard capillary electrophoresis (ABI 3130). The H2D assay is similar in configuration with the exception that the biotin is conjugated to the same antibody (Ab1) that recognizes the same epitope as the VeraTag reporter antibody conjugate (Ab1) as shown in Figure 1(b).

### 6.2. HERmark Assay Workflow

**Figure 2:**
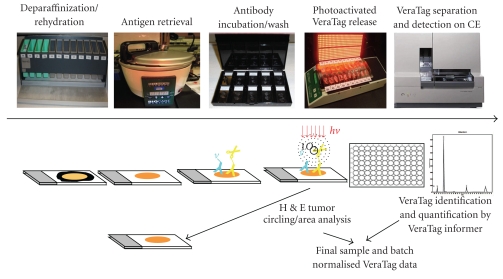
*HERmark Assay Workflow.* Deparaffin/rehydration, antigen retrieval, and overnight antibody incubation steps are performed on Day 1. On day 2, the tumor tissue is incubated with the photosensitizer molecule and illuminated to release VeraTags. VeraTags are collected and separated on capillary electrophoresis and the tumors are H & E stained, tumor area identified and circled and the final sample and batch normalized data is typically available within a 7 day turnaround time.

The HERmark assay workflow has been described in detail in the Material and Methods and is depicted schematically in Figure 2. In brief, the FFPE tumor tissues are subjected to deparaffinization/rehydration, antigen retrieval, and the conjugated antibodies are then added and incubated overnight. The following day the tissues are processed as described in Materials and Methods and then illuminated on a chiller block for two hours. Following illumination, the VeraTag reporters are collected from the tumor specimens and run on capillary electrophoresis and quantified using proprietary informer software. The sections are then H & E stained and the invasive tumor area identified by a board certified pathologist and quantified using an image analysis system as described in Materials and Methods. Tumors that are morphologically more than 50% DCIS are excluded from the analysis. The tumor area is calculated by the identification and circling of the invasive tumor by a board certified pathologist and subsequently quantifying this area using an image analysis system as described in Materials and Methods. The final VeraTag data reported is normalized to the actual tumor area and the entire batch is normalized using cell line controls that have been previously characterized with expected values (data not shown).

### 6.3. Accuracy (Comparison with Known Reference Methods)

**Figure 3:**
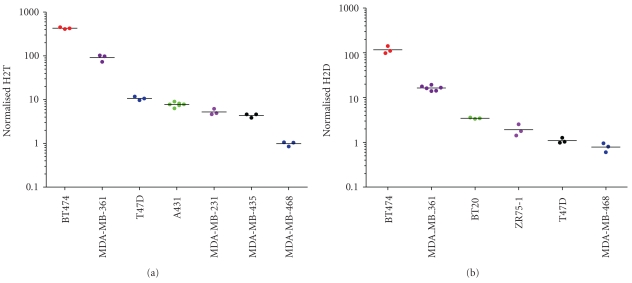
*Accuracy of the H2T and H2D assays.* Seven ((a); H2T) and six ((b); H2D) different cell lines with varying levels of HER2 total protein, as measured by in house ELISA and flow cytometry, were run in the H2T assay in two separate batches. All signals showed correct rank order and accuracy based on ELISA and flow cytometry comparisons.

The accuracy of the H2T assay was assessed by testing multiple independent cell lines that were previously characterized in the literature or in house using cross-validating technologies. HER2 total levels in human cancer cell lines have been published using a variety of analytical techniques, including Western Blot data [27] and ELISA [28]. Minor discrepancies between different published datasets are often attributed to minor changes in how the cells were cultured and harvested and/or the assay used to generate the data. For these reasons, Monogram generated internal datasets on HER2 total levels by both ELISA and flow cytometry (Table 1). The cell lines chosen spanned the range of known HER2 expression from HER2 negative to HER2 3+ as defined by subjective criteria for IHC [29]. For the H2T assay, accuracy was assessed by measuring the HER2 total levels in seven cell lines (BT474, MDA-MB-361, T47D, A431, MDA-MB-231, MDA-MB-435 and MDA-MB-468) selected to span the approximate dynamic range of the assay. Two batches were required to run all 21 samples (3 samples for each of 7 cell lines). Three samples of each of the following four cell lines were run in one batch: BT474, MDA-MB-361, T47D, and A431. The second batch contained the three remaining cell lines (MDA-MB-231, MDA-MB-435, and MDA-MB-468) as well as repeated samples of A431. 100% (21/21 pairwise comparisons) of overall accuracy results matched expected results (Figure 3(a)) in that no overlap was observed between signal levels for any of the seven cell line samples, that is, each cell line separated completely from its nearest neighbor(s). For the H2D assay, no independent measurement of homodimers is currently available, so results were compared to in-house previous H2D assay measurements as well as independent HER2 total measurements [23]. Samples from six different cell lines were each tested in triplicate (BT-474, MDA-MB-361, BT-20, ZR-75-1, T47D, and MDA-MB-468) over two separate batches and 100% (18/18 pairwise comparisons) of the results matched predicted H2D levels (Figure 3(b)) with rank order preservation and no overlap of values. These results suggest that the data from the H2T and H2D assays are comparable to results from ELISA and flow cytometry assays performed concurrently as well as published results [27].

### 6.4. Precision (Intra-Assay Variability) Of the HERmark Assay

**Figure 4:**
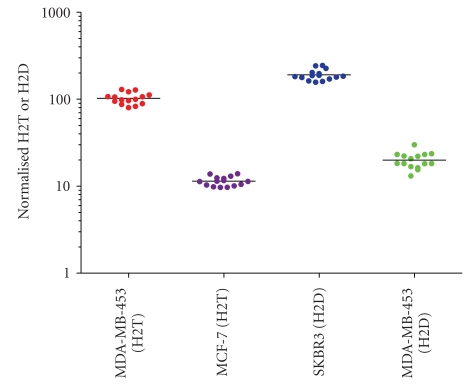
*Precision of the HERmark assay.* Fifteen replicates of each of 2 different cell lines spanning the dynamic range of the H2T and H2D assays were run in one batch for each assay to determine intra-assay variability. 95% of the values in the H2T and H2D assay are within 1.45-fold and 1.65-fold, respectively.

Assay precision was evaluated by determining the variability within a single assay or batch. Measurements were done by testing two independent cell lines spanning the high and low end of the assay dynamic range with 15 replicates per cell line per batch (MDA-MB-453 and MCF-7 for H2T; SKBR3 and MDA-MB-453 for H2D) and analyzing the results using pairwise comparisons. For the H2T assay, 100% (210/210) pairwise comparisons were within 1.7-fold and >95% of the pairwise comparisons were within 1.45-fold (Figure 4). For the H2D assay 100% (210/210) of the pairwise comparisons were within 2.3-fold and >95% of the pairwise comparisons were within 1.65-fold (Figure 4). The %CV for precision of all data in the H2T assay was 14.9% (MDA-MB-453) and 12.5% (MCF-7). The %CV for precision of 100% data in the H2D assay was 14% (SKBR3) and 21% (MDA-MB-453).

### 6.5. Reproducibility (Interassay Variability) of the H2T and H2D Assay

**Figure 5:**
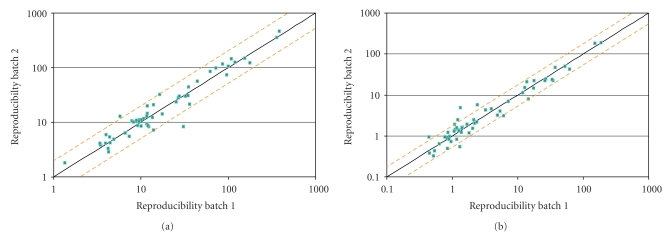
*Reproducibility of the H2T and the H2D assay.* Run 1 and run 2 represent a combination of 45 breast cancer tumors and 11 cancer cell lines assayed in two different batches. The batches were run to incorporate the following comparisons: separation by 10 days, multiple illuminators, chiller blocks, pressure cookers, and capillary electrophoresis instrument, two runs with separate lots of critical control slide reagents and critical liquid reagents. Pairwise comparisons were made on each of the separate runs. For the H2T assay 2 separate runs (a) were performed. For the H2D assay 3 separate runs were performed; results from a typical pairwise comparison are shown in (b).

Assay reproducibility was evaluated by determining the variability between batches. The interassay reproducibility was evaluated over numerous parameters typically encountered in the clinical reference lab, including at least two operators, multiple instruments (illuminators/chiller blocks, scanners and capillary electrophoresis instruments), two lots of critical reagents, two lots of cell line controls, and multiple days/weeks. Samples consisted of a combination of 45 patient-derived FFPE samples (sample IDs not shown) and 12 cell line samples as shown in (Table 2). Samples were grouped into three tumor sets (TS): A, B, and C (TSA, TSB, TSC) and one cell line panel (CLP). Results were compared pairwise after each sample was run in separate batches. For the H2T assay, each sample set was run in two different batches and, for the the H2D assay, each sample set was run in three different batches. Of the reportable values, 96% of the pairwise comparisons were within 2-fold in the H2T assay (Figure 5(a)). For the H2D assay, 95% of the pairwise comparisons were within 2.2-fold (Figure 5(b)). 

### 6.6. Sensitivity of the H2T and H2D Assays

**Figure 6:**
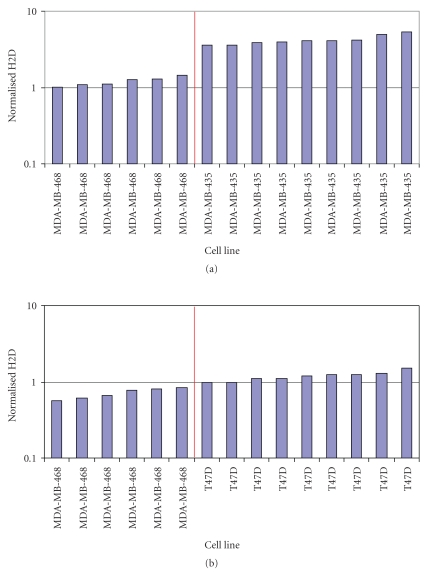
*Sensitivity of the H2T and H2D assays.* (a) To assess the sensitivity of the H2T assay 2 different cell lines were utilized, a negative HER2 total protein cell line, MDA-MB-468, and a low HER2 receptor/cell MDA-MB-435 (IHC score of 0, see Table 2). (b) Sensitivity of the H2D assay was assessed using the negative cell line MDA-MB-468 and the low receptor cell lineT47D (IHC score of 0/1+, see Table 2).

For the H2T assay, two cell lines (i.e., MDA-MB-435 and MDA-MB-468) were selected for sensitivity and the samples run in one batch. The batch consisted of six samples of MDA-MB-468 and nine samples of MDA-MB-435, yielding 54 potential pairwise comparisons. MDA-MB-468 has little or no HER2, while MDA-MB-435 has low signal levels, less than the lowest assay control, MCF7 [27, 28] as well as in house data (not shown). 100% (54/54) of the pairwise comparisons were within the stated acceptance criteria that all MDA-MB-435 values are greater than MDA-MB-468 values. Results are detailed in Figure 6(a), sorted from lowest signal to highest signal. Because the largest MDA-MB-468 signal (1.46) was less than the smallest MDA-MB-435 signal (3.59), detailed pairwise comparison was unnecessary. For the H2D assay, two cell lines (T47D and MDA-MB-468) were selected for sensitivity and the samples were run in one batch. The batch consisted of six samples of MDA-MB-468 and nine samples of T47D, yielding 54 pairwise comparisons. MDA-MB-468 has little or no HER2, while T47D has signal levels comparable to the low control, MCF7 [27, 28], and in, house data, (not shown). 100% (54/54) of the pairwise comparisons were within the stated acceptance criteria (T47D > MDA-MB-468). Results are detailed in Figure 6(b), sorted from lowest signal to highest signal. Since the largest MDA-MB-468 signal (0.82) was less than the smallest T47D signal (0.97), detailed pairwise comparison was unnecessary.

### 6.7. Linearity of the H2T and H2D Assays

The ability of the capillary electrophoresis instrument to evaluate patient samples over a wide range of H2T and H2D levels was demonstrated using the HER2 positive cell line control BT474. Samples were processed according to standard procedures through illumination and conversion as described in Materials and Methods. Following sample dilution, three replicate plates were made and then one plate each was run on three independent capillary electrophoresis instruments. Final reportable values were multiplied by the corresponding dilution factor and then compared in pairwise fashion. Two types of dilutions were performed: serial dilution and nonserial dilution. Serial dilutions were performed to cover the dynamic range of the assay from the highest reportable signal (undiluted) to the lowest reportable signal. Non-serial dilutions were performed to compare from high to low signal in a single dilution. Pairwise comparisons of concentration-corrected signals from the serial dilutions and non-serial dilutions from all three capillary electrophoresis instruments were analyzed in aggregate.

For the H2T 95% (303/319) of the pairwise comparisons were within 1.4-fold (data not shown). For the H2D assay, 95% (183/193) of the pairwise comparisons were within 1.8-fold (data not shown).

### 6.8. Sample FFPE Section Size Validation Testing and Results

The ability of the assay to evaluate patient samples over a wide range of H2T and H2D levels and to confirm that the reportable normalized value is independent of section size was demonstrated using HER2 positive cell line controls, selected to cover the dynamic range of the assay. Sample slides from SKBR3, MDA-MB-361 and BT20 cell lines were each subsectioned to the approximate following sizes: 1 (full), 0.5 (half), 0.25 (quarter), and 0.125. The approximate size of a full section was 50 mm^2^. The slides were prepared by cutting away material from a full slide. For each cell line, two samples of each size were run using in the H2T and H2D assay, except for SKBR3 full size, which was run only once (in each assay) due to batch size constraints. Final reportable values (i.e., after area normalization) were compared in pairwise fashion. 

**Figure 7:**
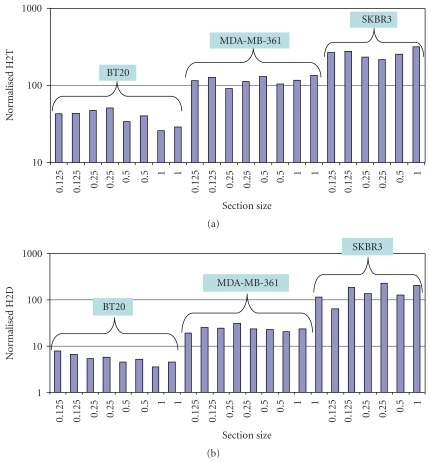
*Section size linearity of the H2T and H2D assays.* (a) Cell line controls with varying levels of H2T (a) and H2D (b) were sub-sectioned and then run in the HERmark assay to determine linearity of the measurements with respect to section size. Normalized values (*y*-axis) show the final signals that have been corrected for size. 95.8% of the pairwise comparisons were within 1.7-fold in the H2T assay and 95% of the pairwise comparisons were with 2.2-fold for the H2D assay.

For the H2T assays, 100% of the pairwise comparisons were within 2-fold and 95.8% of the pairwise comparisons were within 1.7-fold (Figure 7(a)). For the H2D assay, 95% of the pairwise comparisons were within 2.2-fold (Figure 7(b)).

### 6.9. Specificity

**Figure 8:**
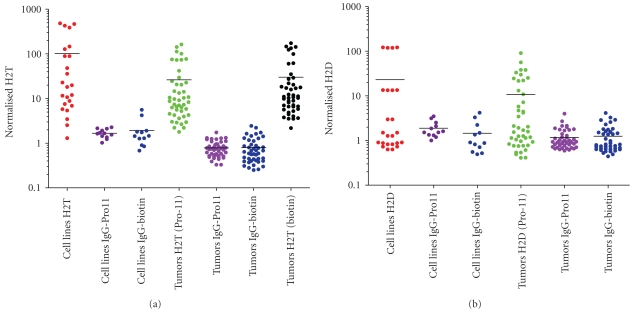
*Isotype control specificity of H2T and H2D assays.* All 44 tumors and 11 cell lines were tested in the regular H2T assay. Slide samples from adjacent sections were also run in an H2T assay where the anti-HER2-pro11 antibody conjugate was replaced with an IgG_1_-pro11 conjugate (“IgG_1_-pro11”) or where the anti-HER2-biotin antibody conjugate was replaced with an IgG_1_-biotin conjugate (“IgG_1_-biotin”). (a) is a graphical representation of the non-specific contribution of signal in the H2T assay whereas (b) represents the non-specific contribution of the signal in the H2D assay.

Assay specificity was demonstrated in a series of experiments designed to assess the frequency of false positive results from experiments using isotype control antibodies as well as false negative results due to the presence of commonly encountered interfering substances. 45 patient-derived tumor samples (data not shown) and 12 cell line samples (Table 2) were run using the H2T and isotype control antibodies. Isotype control antibodies were matched to the constant regions of the respective HER2 antibodies. For the HER2 Ab8-Pro11, the matched isotype control was IgG_1_-Pro11. For the HER2 Ab15-biotin, the matched isotype control was also IgG_1_-biotin. Signal generated from these reactions is not antigen specific, and represents nonspecific background. Samples were run in each of the following three formats for H2T and each format was run in a separate batch: Format 1: HER2 Ab8-Pro11/HER2 Ab15-biotin (assay specific format), Format 2: HER2 Ab8-Pro11/IgG1-biotin, and Format 3: IgG_1_-Pro11/HER2 Ab15-biotin. For H2D, the formats were: Format 1: HER2 Ab8-pro11/HER2 Ab8-biotin (assay specific format), Format 2: HER2 Ab8-pro11/IgG_1_-biotin, and Format 3: IgG_1_-pro11/HER2 Ab8-biotin. Sample results from each IgG_1_ format (Format 2 and Format 3) were compared to the negative control, MDA-MB-468, present in each batch. Samples results were also compared to the respective actual H2T or H2D signal. The results of the H2T assay demonstrated that 100% (112/112) of the results passed this criterion (either within 2-fold of the MDA-MB-468 background signal or less than or equal to 10% of the corresponding H2T signal). Results are shown in Figure 8(a). In the H2D assay, 99% (108/109) of the comparisons were within the stated acceptance criteria (either within 3-fold of the MDA-MB-468 background signal or less than or equal to 20% of the corresponding H2D signal). Results are shown in Figure 8(b).

### 6.10. Interfering Substances Validation Testing and Results

**Figure 9:**
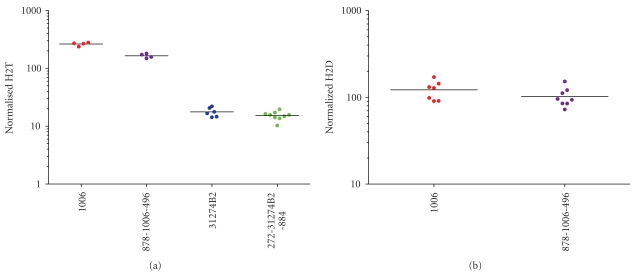
*Tissue Specificity testing of the H2T and H2D assays.* Tissue specificity was determined by measuring the H2T signal (a) or H2D signal (b) generated by the comparison of tumor tissue alone and with tumor tissue in addition to normal fat and stroma. 1006 = breast tumor; 878-1006-496 = breast tumor embedded with stroma and fat (a, b). 31274B2 = tumor; 272-31274B2-884 = breast tumor embedded with stroma and fat (a).

To analyze the effect of interfering substances on the H2T signal, two tumor samples positive for HER2 878/1006/496: 1006—high HER2; 272/31274B2/884: 31274B2—low HER2) were re-embedded with potential interfering substances from normal stroma tissue (496 and 884) and normal fat tissue (878 and 272). The blocks were then cut into slides and a subset of slides was sub-sectioned to remove the normal stroma and normal fat (tumor only: 1006 or 31274B2). These slides were then run with the corresponding whole (i.e., not sub-sectioned) re-embedded slides in the H2T assay. A pairwise comparison of final results was then obtained. For 878/1006/496, four samples of tumor only and four samples of tumor/stroma/fat were run in one batch, resulting in 16 pairwise comparisons. For 272/31274B2/884, six samples of tumor only and 9 samples of tumor/stroma/fat were run in one batch, resulting in 54 pairwise comparisons. A total of 70 pairwise comparisons were obtained and 100% (70/70) of the pairwise comparisons were within the stated acceptance criteria (2.5-fold). Further, 97% of the pairwise comparisons were within 2-fold, and 95% of the pairwise comparisons were within 1.9-fold. Results are detailed in Figure 9(a). 

To analyze the affect of interfering substances on the H2D signal, the same tumor sample as above was used (878/1006/496) and the blocks cut into slides and a subset of slides was sub-sectioned to remove the normal stroma and normal fat. These slides were run with the corresponding whole (i.e., not sub-sectioned) re-embedded slides in the H2D assay. A pairwise comparison of final results was then obtained. Seven samples of tumor only and eight samples of tumor/stroma/fat were run in one batch, resulting in 56 pairwise comparisons. 100% (56/56) of the pairwise comparisons were within the stated acceptance criteria (3-fold). 100% of the pairwise comparisons were within 2.4-fold and 95% of the pairwise comparisons were within 2-fold. Results are shown in Figure 9(b).

### 6.11. Validation Summary

The summary of the HERmark CLIA validation results is shown in Table 3. For each assay, H2T and H2D, several parameters of assay performance were tested including accuracy, precision, reproducibility, sensitivity, linearity, and specificity. The percentage of results passing the validation is based on a 2-fold cutoff where applicable. For the H2D assay, there are two parameters, reproducibility and FFPE section size linearity that did not pass the 95% 2-fold cutoffs and but were 91.8% and 92.2%, respectively. Differences in the amount of H2D in the cell relative to H2T and/or the differences between the two assay formats may account for the decreased performance of the H2D assay relative to the H2T. In addition, the measurement on smaller section sizes may result in variability outside the linear range of the assay.

## 7. Discussion

We described the analytical validation of the HERmark assay that accurately, reproducibly, and sensitively, measures HER2 total protein as well as HER2:HER2 homodimers in FFPE specimens from breast tumor specimens and is based on the VeraTag technology. The relationship between quantitative HER2 expression as measured by HERmark and clinical outcome of metastatic breast cancer patients was previously reported and describes a continuum of HER2 expression that correlates with outcome following trastuzumab treatment. [30, 31]When HERmark measurements are compared retrospectively with IHC and ISH testing on a set of metastatic breast cancer tumors originally classified as HER2 positive and eligible for Trastuzumab treatment, a subset (~13%) was subsequently reclassified [32]. These patients experienced times-to-progression (TTP) following trastuzumab treatment, that were indistinguishable from those of patients classified as central FISH-negative, and significantly shorter TTP than similar FISH- positive patients who also showed over-expression of HER2 by HERmark. When samples were compared retrospectively in larger adjuvant clinical trial (FinHer) originally selected for HER2 status by a combination of local IHC and central CISH, ~13%–23% of patients were reclassified by HERmark depending on the comparison (local or central IHC, or both central IHC and CISH) [33]. Excluding the equivocal cases in this study, HERmark demonstrated 97% concordance with IHC for positive and negative assay values. We have previously established a clinical cut-off whereby patients above a certain threshold of HER2 protein, as determined by HERmark, respond better in time to progression to Trastuzumab treatment than those below this cut-off determined by positional scanning [32]. Interestingly, this clinical cutoff falls within the HERmark equivocal zone which is defined by the 95% confidence of this measurement as compared to ~1090 breast cancer tissue specimens tested by reference methods (IHC/ISH). It is worthwhile to note that the HERmark equivocal category encompasses a relative narrow range (~0.2 log) within the wide dynamic range (~2-3 log) of HER2 distribution, while the semi-quantitative IHC 2+ category (considered equivocal) may include tumors with wider range of HER2 expression. The current IHC testing is based on a subjective, semi-quantitative scale (0, 1+, 2+, and 3+) requiring microscopic evaluation by a board certified pathologist. Standardization of such a method across laboratories requires robust methods to ensure that there is consistency in the testing method as well as the scoring based on a nonautomated system [21]. The HERmark assay provides a continuous, quantitative, and reproducible measurement for H2T and H2D over a dynamic range of ~3 logs and ~2 logs, respectively. This allows the accurate separation of cell line controls that were originally classified as 2+ to 3+ into a continuum that spans a HER2 protein levels 5–10-fold as measured by HERmark. These results agree quite well with in-house measurements using low throughput quantitative methods such as flow cytometry and ELISA and testing on the same cell line preparations as used for the FFPE blocks. We have also demonstrated previously that H2T measurement in a set of 170 tumors correlates well with in-house IHC measurements [23]. Furthermore, the corresponding histoscores (H-score), on this subset of tumors, as measured by HER2 IHC, demonstrate a plateau in the IHC 3+ category and the inability to accurately stratify at high levels of HER2 protein, whereas measurement by H2T demonstrates>10-fold difference in dynamic range [23]. 

There is no standard way to measure HER2 homodimer levels but it has been demonstrated previously that overexpression of HER2 protein expression may be required to transform a cell line *in vitro* [2]. Biologically a difference in HER2 receptor levels in the range of 5–10-fold may have significant impact on patient response to Trastuzumab [34]. This is especially important when considering the low-response rates and emerging evidence that levels of p95, the truncated form of full-length HER2, correlate with higher levels of H2T [34]. In addition to the extension of the dynamic range at the higher end, the HERmark assay is able to accurately distinguish a set of cell line controls classified by IHC as 0 to 1+ but spanning a ~20 fold dynamic range from a negative control cell line, MDA-MB-468 that has no detectable HER2 protein expression as measured by ELISA. This difference corresponds to a range of HER2 total receptors per cell from low/negative up to ~30,000 as determined by flow cytometry. We have demonstrated that the sensitivity of the H2T measurement is equivalent to 2500 receptors/cell whereas conventional IHC is typically 7–10 times less sensitive. Therefore, when the cell line MDA-MB-435 is measured by IHC the result is a classification of 0 or negative (in house data). Recently, adjuvant clinical trials have indicated that even patients that are not overexpressing HER2 as measured by IHC and FISH, may respond to Trastuzumab treatment, indicating that accurate and sensitive measurements at the lower end of the dynamic range may also be clinically relevant [16]. Similarly, the quantitative measurement of low levels of HER2 may be clinically relevant for other types of solid tumors as in the case of Barrett's esophagus-associated adenocarcinoma cancer [35]. Other quantitative measurements of HER family proteins (EGFR and HER2) have been developed based on the AQUA technology, however, in the case of HER2 it has been shown that two different concentrations of HER2 antibody are required to accurately quantify the HER2 levels depending on whether the measurements are made in the upper range or lower dynamic range of expression [36]. Applying this to routine practice could be quite cumbersome and may increase the subjective nature of the HER2 protein measurement. The HERmark assay consists of cell line controls that span the entire dynamic range of HER2 protein expression and are used to control for batch to batch variability, allowing comparisons to be made over time between clinical samples. Since a patient sample is measured only once, it was important to rigorously test the intra- and interassay variability to determine the confidence level surrounding this measurement. Precision experiments demonstrated that if a patient sample is measured multiple times within a batch, there is a 95% probability that the H2T value will be within 1.45-fold and that the H2D value will be within 1.65-fold. The precision was determined using cell lines that span above and below the clinical cutoff for response to Trastuzumab in a test cohort as decribed above. With a dynamic range of 2-3 logs, the potential of classifying a patient as a false positive or negative theoretically should be greatly reduced; however, there will be some ambiguity in the measurement at or around the clinical cutoff based on the analytical performance of the assay. This analytical performance applies to all methods of HER2 testing including IHC, ISH, and mRNA measurements and may explain why there is so much reclassification when standard testing is compared to HERmark, especially in the equivocal category. Inter assay reproducibility was evaluated under different parameters typically encountered in a clinical laboratory including different operators, multiple instruments, different days and two lots of critical reagents. This also allows the comparison of what might be typically associated when samples are run in different central labs as routinely done for HER2 IHC and FISH testing for Trastuzumab inclusion. In addition to good reproducibility the specificity of the assay is increased due to the fact that the VeraTag technology relies on the binding of two epitope-specific antibodies in close proximity. Conventional IHC relies on a single antibody and requires high degree of specificity that is difficult to achieve with varying heterogeneity in tumor samples. Batch normalization of the data allows for Meta analysis of clinical studies and to validate potential clinical cutoffs as well as to monitor assay performance over time. In general, a higher concordance was observed between H2T by HERmark and HER2 assessed by more stringent central testing as compared with local HER2 testing by IHC in that the results were 97% concordant overall (excluding equivocals) [37]. Furthermore, HERmark positive breast cancers were significantly associated with invasive ductal carcinoma, high tumor grade, estrogen/progesterone receptor negativity, and expression of Ki67 [38]. Thus the quantitative HER2 measurement by HERmark confirms the known correlations between HER2 expression and clinical pathologic characteristics of breast cancer. The use of a more accurate and quantitative HER2 measurement may allow better stratification of patients for response to HER2 targeted therapies as well as improve the accuracy and sensitivity of the testing methodology.

## Figures and Tables

**Table 1:** *Accuracy of cell line controls as measured by flow cytometry, IHC, and ELISA.* The majority of the cell lines used for accuracy studies are of breast origin with the exception of MDA-MB-435 which has been shown to be of melanoma origin and A431, which is from an epidermoid carcinoma of the vulva. All cell lines were grown in-house and FFPE blocks prepared on the same day as lysates prepared for subsequent HER2 ELISA assay. The flow cytometry was performed on the same day that the cells were fixed and made into blocks. The ELISA experiments represent an *n* = 3 experiments performed on separate days. The flow cytometry assays were done in triplicate on the same as described in materials and methods.

Cell Line	HER2 IHC Score	Flow Cytometry HER2 (receptors/cell)	Flow Cytometry %CV	ELISA [HER2] ng/mg	ELISA %CV
BT474	3+	499795.2	10.4	295.5	15.9
MDA_MB_361	2/3+	212456.0	1.9	65.7	7.4
BT-20	N/A	64,386	3.6	15.5	13.0
ZR-75-1	1+	63,708	2.2	22.1	6.4
T47D	0/1+	32303.1	8.2	16.3	11.3
A431	0	27343.1	0.8	23.3	2.3
MDA_MB_231	0	11249.6	21.5	5.1	43.7
MDA_MB_435	0	2420.5	6.1	2.9	70.7
MDA_MB_468	0	10567.1	17.0	1.0	na

**Table 2:** Summary of the cancer cell lines (A) used in the validation of the HERmark assay.

Cell Line	Source	Source ID
SKBR3	ATCC^(1)^	HTB-30
BT20	ATCC	HTB-19
MDA-MB-453	ATCC	HTB-131
MCF7	ATCC	HTB-22
MDA-MB-468	ATCC	HTB-132
BT474	ATCC	HTB-20
MDA-MB-361	ATCC	HTB-27
T47D	ATCC	HTB-133
MDA-MB-231	ATCC	HTB-26
MDA-MB-435	ATCC	HTB-129
ZR-75-1	ATCC	CRL-1500
A431	ATCC	CRL-1555

^(1)^ATCC= American Type Culture Collection.

**Table 3:** H2T and H2D assay validation summary. The parameters tested in the validation of the HERmark assay are summarized and the percentage passing is based on a 2-fold cutoff. NA indicates that a detailed pairwise comparison was not necessary and so therefore the 2-fold cutoff was not applicable. ^1^95th percentile at 2.2 fold cutoff.

Parameter	H2T Summary	% Within Two-fold Cutoff	H2D Summary	% Within Two-fold Cutoff
Accuracy	Pass	NA (100)	Pass	NA (100)
Sensitivity	Pass	NA (100)	Pass	NA (100)
Precision	Pass	100	Pass	99.5
Reproducibility	Pass	96.4	Pass	91.8^1^
Linearity: CE	Pass	99.7	Pass	99%
Linearity: Section Size	Pass	100	Pass	92.2^1^
Specificity: Triple Re-embed	Pass	97.1	Pass	94.6
Specificity: Isotype	Pass	NA (100)	Pass	NA (99.1)
